# Clinical and Molecular facets of Dengue Virus infection from Bengaluru, South India

**DOI:** 10.3126/nje.v11i3.37712

**Published:** 2021-09-30

**Authors:** Shantala Gowdara Basawarajappa, Ambica Rangaiah, Shwetha Jinnahalli Venugopal, Chakrakodi N Varun, Vijay Nagaraj, Shashiraja Padukone, Sathyanarayan Muthur Shankar

**Affiliations:** 1 Department of Microbiology, Bangalore Medical College and Research Institute, Bengaluru, Karnataka, India; 2 State Level VRDL, Department of Microbiology, Bangalore Medical College and Research Institute, Bengaluru, Karnataka, India; 3 Institute of Animal Health and Veterinary Biologicals, Hebbal, Bengaluru-560024

**Keywords:** Demography, Dengue Virus, Enzyme-Linked Immunosorbent Assay, Genotype, Real-Time Polymerase Chain Reaction

## Abstract

**Background:**

Dengue virus (DENV) continues to be an epidemic with high mortality rates. The clinical features, especially in the early phase of infection, are nonspecific and there is no single marker that can be reliably deployed for diagnostics. Further, serotype and genotype diversity is not clearly understood. This study was conceived to understand the performance characteristics of various diagnostic markers; serotype and genotype distribution is thus a vital requirement.

**Methods:**

A subset of blood samples was obtained for all the clinically suspected Dengue cases during the period January to December 2017. The samples were tested for IgM and IgG antibodies and NS1 antigen by both ELISA and rapid tests. Real-time PCR, Conventional PCR and sequencing was performed based on the serology results. Correlation of the data with demographic and clinical details was used to analyze the performance characteristics of various tests.

**Results:**

Clinical signs and symptoms could not predict dengue positivity due to lack of specific symptoms. The performance of IgM rapid test was found to be lower than the ELISA method (53.5% agreement). The NS1 rapid and NS1 ELISA tests were comparable (89.2% agreement). Majority of the infections were caused due to DEN-2 serotype and phylogenetic analysis revealed all the sequenced DEN-2 serotypes belong to Genotype IV. Three sequences were deposited into NCBI GenBank (GenBank accession number MW583116, MW579054 and MW579053).

**Conclusion:**

Our comprehensive data suggests that NS1 ELISA and PCR are best used in the early phase of dengue infection (< 5 days post-onset of fever), whereas IgM antibody detection is reliable only in the late phase. We also highlight the unreliable performance of rapid tests.

## Introduction

Dengue fever represents the most crucial mosquito mediated arboviral infection affecting several tropical and subtropical regions of the world. Dengue Hemorrhagic Fever and Dengue Shock Syndrome are caused by the Dengue Virus, a single-stranded positive-sense RNA virus belonging to the Flaviviridae group of genus Flavivirus [1]. The current global burden of dengue is estimated to be over 40% with about 2.5 % of the cases resulting in mortality [2]. Dengue fever is caused by all four serotypes (DEN 1-4). All four serotypes have been reported from India [3-6]. The dengue virus is currently prevalent across India as reported by the National Vector Borne Disease Control Programme (NVBDCP).

Diagnosis of Dengue fever in the clinical laboratory has proven to be complicated, especially since the clinical symptoms are often non-specific. The specific clinical signs such as retro-orbital pain, petechiae and rash accompanied by febrile illness are seen only in the late stages [7]. Further, Dengue IgM detection assays have a shallow predictive value in the early phase of dengue fever [8]. In contrast, NS1 detection assays and PCR based testing have a better sensitivity [9,10]. Currently, there are no dengue specific antivirals available and hence treatment is mostly symptomatic management [11,12].

Attempts have been made with regard to development of algorithms based on clinical features and laboratory techniques in the diagnosis of dengue [7-10]. However, there is a lacuna in data comparing both clinical and laboratory diagnosis in a single study. In this study, we attempted to capture the importance of clinical signs and symptoms, various laboratory tests and molecular analysis in context of dengue infection in a single study.

## Methodology

### Study design and participants

Clinically suspected dengue cases that were referred to State Level Virus Research and Diagnostic Laboratory (VRDL), Bangalore Medical College and Research Institute (BMCRI), Bengaluru, India during the period January to December 2017 formed the population of this study. Blood samples were collected in a labelled vacutainer tube (SST advanced; Cat no. BD 367954; Beckton Dickinson, USA) and transported to the laboratory within 30 min of collection. The serum was collected and stored at 4°C, for up to 48 hours. The samples were stored for a long term in -80° C, in multiple vials and were thawed for testing only once.

### Data Collection

For feasibility, a subset of the sample (n=331) was subjected to further testing by serology and Real-time PCR. Demographic and clinical details including duration of illness, fever, nausea, vomiting, rash, abdominal pain, myalgia, arthralgia, headache, retro-orbital pain, bleeding and previous history of infection were collected for all cases. The clinical signs and symptoms were used to construct a regression model.

### Serological testing

All the serum samples were tested by IgM MAC ELISA (NIV, Pune), NS1 ELISA (Panbio; USA) and Dengue Day 1 test (J Mitra and Co., India) as per the manufacturer’s protocol [13-15]. In addition, IgM MAC ELISA positive cases were also analyzed by IgM ELISA (Panbio; USA) and IgG ELISA (Panbio; USA) as per the manufacturer’s protocol [16]. The IgG/IgM ratio (Cut off value= 1.10) was used to determine the case as primary or secondary dengue based on previously published literature [17].

### RNA extraction and Real time PCR analysis

All samples that tested positive or equivocal either by IgM MAC ELISA or NS1 ELISA, was further tested by Real time PCR (RT PCR). The viral RNA was extracted from the serum sample using QIAampViral RNA Mini kit (Qiagen, Germany). Real time PCR was performed using CDC DENV-1-4 rRT-PCR multiplex assay kit in CFX96 Touch Real-Time PCR Detection System (BioRad; USA) as previously described [18, 19].

### Conventional PCR and Sequencing

The extracted RNA was subjected to reverse transcriptase PCR. Conventional PCR was performed based on the previously described protocol [20]. The details of the primer sequences are provided in Table 2. The reverse transcriptase PCR testing was performed in Master Cycler Nexus gradient instrument (Eppendorf; US). In brief, cycling conditions were as follows. Initial denaturation was performed at 95°C for 10 min. This was followed by 35 cycles of denaturation at 95°C, annealing at 60°C and extension at 72°C each for 30 seconds. The final extension was performed at 72°C for 5 min. The PCR products were visualized in 2% agarose gel.

The PCR gel clean up and sequencing was performed at a commercial setup (Eurofins; Bengaluru). The PCR product was sequenced by Sanger sequencing method. Sequence quality check and trimming was performed using Finch TV (V 1.4) software. The data obtained were queried using Basic Local Alignment Search Tool (BLAST) and ascertained for sequence identity. The sequences with more than 200 bp upon trimming were submitted into NCBI GenBank to obtain the GenBank accession number.

### Phylogenetic analysis

The DEN-2 sequences obtained from this study were aligned with reference sequences available in NCBI GenBank representing various geographical and genotypes using ClustalW Pairwise - Multiple Alignment using Mega 7 package. The phylogenetic tree was constructed by the Neighbor-Joining method using 1000 bootstrap replicates [21].

#### Outcome variable

Outcome variables included the performance of various diagnostic tests used for dengue detection like percentage positivity, positivity in relation to duration of symptoms, distribution of primary and secondary cases.

#### Explanatory variable

Demographical characteristics such as age, gender, clinical manifestations, dengue serotypes and genotypes.

### Ethical Clearance

The present study obtained approval from the Institutional ethics committee, Bangalore Medical College and Research Institute, Bengaluru (Vide Ref: No.BMCRI/PS/210/2019-20).

### Sample size calculation

Among the clinical samples archived at -80°C, a subset of 331 samples were selected by stratified random sampling matching demographic characteristics like age and gender apart from percentage of IgM positivity of the population under consideration for the study.

### Data management and statistical analysis

All the data obtained for this study was tabulated in Microsoft Office Excel (2007). The statistical analysis was performed using R statistical software (R Project; V 3.6). Frequency distribution was assessed as percentage of the population. For the continuous variables central tendencies were assessed using mean and standard deviation. Shapiro Wilk test was used to assess normality distribution. Comparison of variables of nominal scale was done using chi square test. Clinical symptoms were assessed using multinomial logistic regression analysis using “nnet” package to study the outcome.

## Results

A total of 6126 clinically suspected dengue samples were received for Dengue IgM testing in the State Level VRDL, BMCRI during the year 2017. Of these, 1986 samples (32.4%) were positive for Dengue IgM antibodies by ELISA (NIV kit). Of the 331 samples selected as a subset by stratified random sampling from the population for the purpose of the present study, 105 samples (31.7%) were found to be positive for Dengue IgM antibodies by NIV ELISA kit. The age, gender and sample positivity rate were not statistically significant between the population and sampling subset (X2 >0.05). The clinical and demographic summary of the cases tested in this study is provided in Table 1. Logistic regression model was not successful in predicting dengue infection.

**Figure 1:**
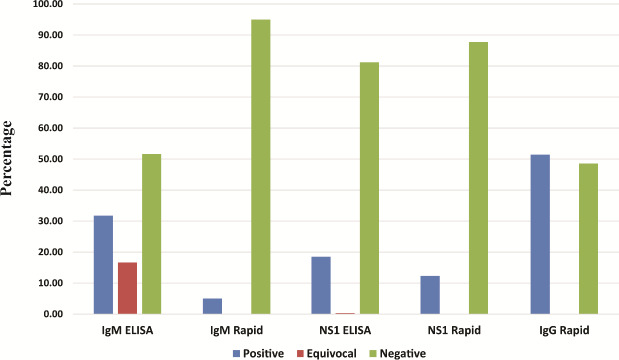
Results of various serological tests (by percentage)

**Figure 2:**
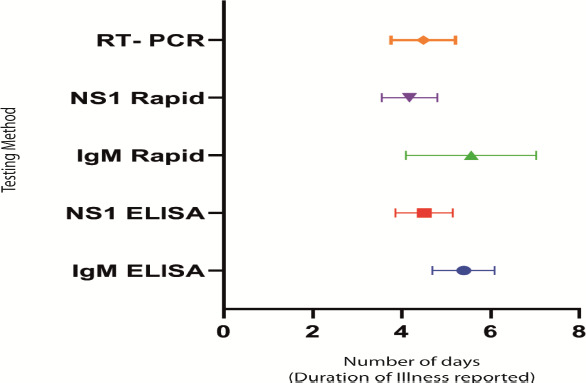
Mean and Standard deviation of tests showing positivity by number of days after onset of fever. The Mean with 95% Confidence interval is shown.

**Figure 3:**
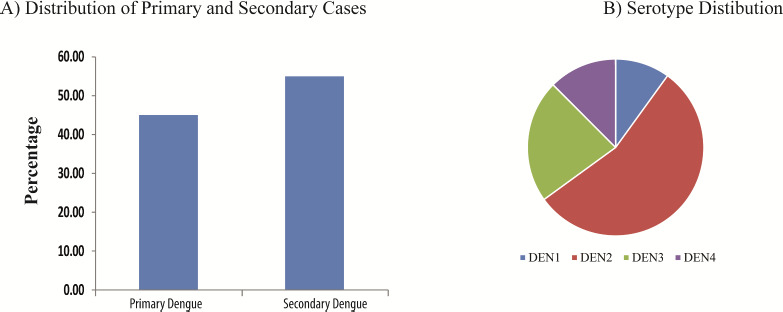
A) Distribution of primary and secondary dengue cases. B) Distribution of Dengue Serotypes as assessed by RT PCR testing.

The IgM ELISA and IgM rapid test was in agreement in only 53.5% cases. In contrast, the concurrence between NS1 ELISA and NS1 Rapid test was found to be higher (89.2%). The concurrence rate was not statistically significant between the genders and was not found to be influenced by age. IgG was found to be positive by rapid test in 48.94% of the cases (Data summarized in Figure 1). NS1 ELISA was found to be positive at 4.5 days (95% CI; 3.87-5.12) of onset of fever. Real time PCR was also found to be positive following 4.48 days (95% CI, 3.77-5.18), similar to NS1 results. IgM ELISA was positive at 5.39 days (95% CI; 4.69-6.08). Figure 2 summarizes the number of days during which the individual tests are found to be positive. IgG/IgM ratio was used to determine the nature of infection as primary or secondary. It was also observed that 43.96 % of the cases were serologically primary dengue and the remaining cases were secondary in nature (Figure 3A).

**Figure 4:**
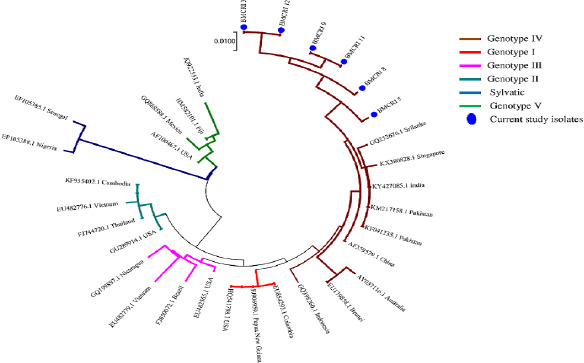
Phylogenetic analysis

A total of 112 samples were tested by real-time RT-PCR of which, 40 samples (35.7%) tested positive. All four serotypes were detected by RT-PCR, with DEN-2 subtype being the most predominant (Fig 3B). Conventional PCR showed positivity only in 13 samples, all of which were sequenced. Analysis of the sequence by BLAST analysis confirmed that all the sequences were from Dengue virus. Out of 13, 6 were DEN-2 serotype, three each DEN-1 and DEN-3 serotypes and 1 DEN-4 serotype; the results were concordant with the RT-PCR findings. Three sequences with more than 200bp length were submitted into NCBI GenBank to obtain the GenBank accession number MW583116, MW579054 and MW579053. The phylogenetic tree constructed for DEN-2 serotype showed clustering of the sequences with Dengue Genotype IV (Figure 4).

5A and 5B is used for detection of DEN1; 5A and 5C is used for detection of DEN2; 5A and 5D is used for detection of DEN3; 5A and 5E is used for detection of DEN4. The PCR is a multiplex reaction which can simultaneously detect DEN1-4.

Phylogenetic analysis was carried out using MEGA 7 software. Neighbor-Joining method was used to predict the evolutionary history with 1000 bootstrap values. The Maximum Composite Likelihood method was used to compute evolutionary distance and is in the units of the number of base substitutions per site. The composition bias among sequences was considered in evolutionary comparisons.

## Discussion

### Clinical characteristics and performance of diagnostic tests

Dengue fever has continued to present itself as a global problem, with a prevalence of approximately 100 million cases reported every year globally. The mortality rate of dengue, though varies by geographical location, has been estimated to cause at least 20,000 deaths a year. The current scenario in India is reflective of the global trend [22]. The diagnostic support for the detection of dengue includes serological detection of NS1 antigen or IgM antibodies by various methods such as rapid card test or ELISA and virus nucleic acid detection [23]. However, the usefulness of these diagnostic markers has been debated. Further, the sensitivity of detection for each assay varies by the duration of illness. NS1 is detectable during the acute phase of dengue virus infections with sensitivity comparable to molecular tests during the first 0-7 days of symptoms. IgM levels start rising in the first week of infection with a peak during the second week followed by a decline. Anti-dengue IgG levels are usually undetectable in the first week after onset of symptoms, gradually rising with detectable levels persisting for months. [23-28].

In the present study, 6126 clinically suspected dengue samples were processed for diagnostic workup, which primarily included a request for IgM detection. Sampling of subset was undertaken for downstream analysis. A total of 331 samples were randomly selected for this study with demographic characteristics like age, gender and percentage of sample positivity by IgM being similar to that of the population, thereby justifying the sampling protocol. Among the cases, male predominance (n=198) was noted. The commonest clinical manifestations noted among the cases in the present study included fever (98.4%), headache (55.5%) and myalgia (51.5%). These clinical parameters were used to construct a logistic regression model which failed to predict dengue status independently. This finding was consistent with previously reported literature [7, 24]. This observation is also probably due to a large number of cases presenting with non-specific symptoms and the average duration of illness being less than seven days, which is not sufficient to develop specific symptoms. [7].

Dengue IgM MAC ELISA (NIV kit) showed a positivity of 31.72%, which was significantly higher than the IgM rapid test (5.05%). This was consistent with previous reports suggesting that the NIV kit has a much higher positivity rate as compared to other kits or methods [13]. Considering RT-PCR as a reference standard, we looked for the impact of reported duration of illness on IgM. It was found that IgM had a significantly increased positivity on an average of 5.26 ± 2.47 days and negative results were higher at 3.38 ± 1.8 days (p= 0.03). This finding was consistent with the existing literature indicating approximately 5 days (considering onset of fever as day 0) is required for the dengue IgM to appear positive by ELISA [29].

Rapid tests are helpful tools as point of care diagnostics. However, their sensitivity and reliability are lower compared to ELISA. Further, the agreement between ELISA and rapid tests vary depending on several factors such as viral load, duration of illness and the kits used. In this study, the agreement between dengue IgM rapid and ELISA was poor. This finding is in concurrence with recent views indicating the unreliability of rapid testing [15]. However, the concurrence between NS1 ELISA and NS1 Rapid test was good, indicating that rapid tests are better reliable when detecting NS1 antigen. The results of these tests were not influenced by gender or age.

All the samples that were either positive by IgM ELISA or NS1 ELISA were tested using RT-PCR. Of the 112 samples that were tested by RT-PCR, 40 samples tested positive (35.7%) for one of the Dengue subtypes. The finding suggests that not all NS1 positive samples are PCR positive. The finding is consistent with the literature evidence indicating that the NS1 peaks during 3^rd^ to 5^th^ day following fever and tend to be detectable for more than a week [30, 31]. Our data concur with the principle that PCR remains positive for less than 5 days (Figure 2). Similar findings have been reported previously [27,32].

These findings have a significant clinical impact. Clinically, NS1 antigen is detectable in the early phase of dengue infection, which is typically less than five days. During this phase, the clinical presentation is non-specific. Since the detection is reliable with rapid testing, bedside rapid tests can serve as a reliable tool. However, the results need to be confirmed by NS1 ELISA and PCR testing. On the contrary, typically after 5 days clinical symptoms are more specific and accompanied by IgM production. In these cases, IgM ELISA based testing is a better diagnostic indicator.

The clinical manifestations observed in dengue fever are due to the immunological reaction. When a subject is infected with dengue for the first time, the infection tends to have limited propensity and a long-term immunity is acquired. However, subsequent infection leads to a stronger immune reaction. Based on this serologically, dengue can be distinguished into primary or secondary. A higher level of IgM characterizes primary dengue as compared to IgG [17]. In this study, a higher frequency of secondary dengue was observed indicating the high burden of dengue virus at our geographic location.

### Serotyping and Genotyping

Molecular monitoring of DENV is important to trace DEN serotypes as well as to identify co-infection with dual or multiple serotypes. In the present study, real-time RT-PCR revealed DEN-2 serotype to be the most predominant serotype, followed by DEN-3, DEN-4 and DEN-1. Conventional PCR was positive only in a subset of RT-PCR positive samples (13 positive by conventional PCR against 40 by real-time RT-PCR) indicating a superior sensitivity of RT-PCR. Co-circulation of DEN viruses noted during the present study is consistent with the findings of earlier studies from various parts of India. [33-37] Chakravarti A et al have reported a changing pattern of DEN circulation in Delhi with DEN-1 emerging as the predominant serotype in 2008 displacing DEN-2 and DEN-3 reported earlier in 2003-04 [38]. Vanlalhmingthanpuii et al have reported DEN-3 type being predominant in Aurangabad, Maharashtra followed by DEN-2. They have also described DEN-2/3 & DEN-2/4 co-infections [39].

## Limitation of the study

1. The study does not analyze data for structural changes that might have occurred due to nucleic acid mutations
2. Limited number of dengue isolates sequenced.

## Conclusion

A comprehensive analysis of dengue suspected cases by several serological and molecular analysis together confirms that no single test can be individually used as a diagnostic marker. The clinical symptoms are not sufficiently distinctive to provide a lead, especially in the early infection. Reporting the number of days post-onset of fever is a valuable tool in deciding the best diagnostic test to be performed. Phylogenetic analysis of the DEN-2 serotype confirmed the existence of Genotype IV, which is the most common dengue serotype prevailing in this region. Molecular monitoring of dengue is recommended for identification of circulating serotypes in a geographic region.

### Future scope of the study

Continuous monitoring of the epidemiology of dengue is required to assess the severity of disease and associated dengue serotype and genotype. Hence, this study would lay down the basement for future epidemiological data and help in understanding the disease trends and mutations.

### What is already known on this topic?

Dengue serotypes and molecular epidemiology studies have been carried out in various parts of India.

### What this study adds

This study helps to understand the local epidemiology of dengue, clinical characteristics, circulating serotypes, and genotypes contributing to Dengue epidemiology and management.

## Figures and Tables

**Table 1:** Demographic and Clinical summary

Parameter	Statistical Summary
**Age**	27.88 ± 15.19 years
**Gender**	133 Female / 198 Male
**Duration of Illness**	5.17 ± 4.28
**Fever**	98.4 %
**Nausea**	14.5%
**Vomiting**	26.5%
**Rash**	0.04%
**Abdominal Pain**	18.7%
**Myalgia**	51.5%
**Arthralgia**	41.9 %
**Headache**	55.5%
**Retro orbital pain**	0.07%
**Bleeding**	0.012%
**Previous history of infection**	0.04%

**Table 2:** Primers Used For Conventional PCR and Sequencing

Sl. No	Oligo Name	Sequence (5’ to 3’)
**1**	5A	AGTTGTTAGTCTACGTGGACCGACA
**2**	5B	CCCCGTAACACTTTGATCGCTCCATT
**3**	5C	CGCCACAAGGGCCATGAACAG
**4**	5D	GCACATGTTGATTCCAGAGGCTGTC
**5**	5E	GTTTCCAATCCCATTCCTGAATGTGGTGT
